# Agreement between functional connectivity and cortical thickness-driven correlation maps of the medial frontal cortex

**DOI:** 10.1371/journal.pone.0171803

**Published:** 2017-03-22

**Authors:** Hyunjin Park, Yeong-Hun Park, Jungho Cha, Sang Won Seo, Duk L. Na, Jong-Min Lee

**Affiliations:** 1 School of Electronic and Electrical Engineering, Sungkyunkwan University, Suwon, Korea; 2 Center for Neuroscience Imaging Research, Institute for Basic Science, Suwon, Korea; 3 Department of Biomedical Engineering, Hanyang University, Seoul, Korea; 4 Department of Neurology, Samsung Medical Center, Sungkyunkwan University School of Medicine, Seoul, Korea; University of Pennsylvania Perelman School of Medicine, UNITED STATES

## Abstract

Parcellation of the human cortex has important implications in neuroscience. Parcellation is often a crucial requirement before meaningful regional analysis can occur. The human cortex can be parcellated into distinct regions based on structural features, such as gyri and sulci. Brain network patterns in a given region with respect to its neighbors, known as connectional fingerprints, can be used to parcellate the cortex. Distinct imaging modalities might provide complementary information for brain parcellation. Here, we established functional connectivity with time series data from functional MRI (fMRI) combined with a correlation map of cortical thickness obtained from T1-weighted MRI. We aimed to extend the previous study, which parcellated the medial frontal cortex (MFC) using functional connectivity, and to test the value of additional information regarding cortical thickness. Two types of network information were used to parcellate the MFC into two sub-regions with spectral and Ward’s clustering approaches. The MFC region was defined using manual delineation based on in-house data (n = 12). Parcellation was applied to independent large-scale data obtained from the Human Connectome Project (HCP, n = 248). Agreement between parcellation using fMRI- and thickness-driven connectivity yielded dice coefficient overlaps of 0.74 (Ward’s clustering) and 0.54 (spectral clustering). We also explored whole brain connectivity using the MFC sub-regions as seed regions based on these two types of information. The results of whole brain connectivity analyses were also consistent for both types of information. We observed that an inter-regional correlation map derived from cortical thickness strongly reflected the underlying functional connectivity of MFC region.

## Introduction

Parcellation of the human cortex yields structurally or functionally distinct sub-regions [1–3]. Structural features, especially sulci and gyri, have been widely used to divide the cerebral cortex into distinct regions [4,5]. The inherent limitation of this approach is that an anatomical boundary cannot fully account for the functional capabilities of a given cortical region. Passingham et al. attempted to parcellate the cortex using the patterns of connection in a given region with respect to its neighbors [6]. They coined the term “connectional fingerprint” to convey that each cortical sub-region has a unique connectivity pattern that distinguishes it from other sub-regions. This approach was successfully applied to parcellate many sub-regions of the human cerebral cortex, including the medial frontal cortex (MFC) [1,2].

Many studies have computed functional connectivity using data from functional magnetic resonance imaging (fMRI) and structural connectivity from diffusion tensor imaging (DTI) to parcellate the cortex [2,7–11]. Connectivity based parcellation (CBP) is capable of revealing fine grained functional sub-regions and has become an important tool in neuroimaging [7]. CBP using resting-state fMRI (rs-fMRI) was applied to parcellate the whole brain and cortical structures such as the supplementary motor area (SMA) and insula [2,8,9]. CBP using diffusion imaging has also been applied to parcellate the whole brain and thalamus [10,11]. Other studies have used morphological features derived from structural MRI, such as cortical thickness, to assess inter-regional morphological correlations [7,8]. CBP results depend on the type of connectivity information fed to the parcellation algorithm. Connectivity information derived from different imaging modalities could be different, and thus CBP using different imaging modalities could differ within a given brain region. CBP using diffusion MRI and rs-fMRI were consistent for the insula [9,12]. We aimed to explore whether CBP using different imaging modalities was consistent for an underexplored brain region. The MFC is a clinically important cortical sub-region that consists of two functionally distinct sub-regions: the supplementary motor area (SMA) and the pre-SMA [1]. The SMA is closely linked to motor control and the pre-SMA is linked with complex cognitive controls [1]. Accurate parcellation of the MFC would allow us to better characterize brain alterations related to motor function and complex cognitive controls. A previous study parcellated the MFC based on DTI- and fMRI-driven connectivity [1]. Another study employed fMRI-driven connectivity to parcellate the MFC [2]. We aimed to extend the previous study which parcellated the MFC using functional connectivity and to test the value of additional information regarding cortical thickness [2].

Connectivity derived from correlation thickness was used to replicate many known neuroanatomical pathways. Thickness-driven connectivity showed “small-worldness,” an important property of functional brain networks [13]. Cortical thickness can provide morphological information about brain regions and thus might provide complementary information not obtainable with DTI [13]. We did not consider diffusion MRI, as combining diffusion MRI and rs-fMRI has already been done [1]. Parcellation was performed using spectral clustering and Ward’s clustering approaches. We computed network information from two imaging modalities for the MFC, one derived from cortical thickness and the other derived from rs-fMRI. We then used that information to parcellate the MFC into two sub-regions. We compared the results of MFC parcellation based on cortical thickness obtained from structural MRI and functional correlation obtained from rs-fMRI. We also explored whole brain connectivity based on these two types of network information using the MFC sub-regions as seed regions.

## Methods

### Subjects and image acquisition

The Institutional Review Board (IRB) of Samsung Medical Center approved this study. All participants in this study provided written informed consent. We considered two sets of data. Data from the first set (n = 12) were used to define the MFC region. Data from the second set (n = 248) were obtained from the Human Connectome Project (HCP), which was released in December 2015 [14–16]. The second data set was used to test parcellation approaches. HCP data were chosen so that the second set of data had a similar age and sex ratios compared to the first set. For the first data set, 12 healthy subjects (6 males and 6 females; mean age ± standard deviation (SD): 22 ± 2 years) were scanned. Participants underwent T1-weighted imaging and Echo Planar Imaging (EPI) at Samsung Medical Center, Seoul, Korea. Images were acquired with a Philips Intera Achieva 3.0 Tesla scanner equipped with an 8-channel SENSE head coil (Phillips Healthcare, the Netherlands). T1-weighted images were acquired using a magnetization-prepared gradient echo sequence with the following image parameters: TR = 9.9 ms; TE = 4.6 ms; flip angle = 8°; 298 slices; with voxel dimension = 0.5×0.5×1 mm^3^. Imaging parameters for the EPI were as follows: TR = 3000 ms; TE = 35 ms; flip angle = 90°; 35 slices; and voxel dimension = 1.72×1.72×4 mm^3^. EPI data consisted of 155 volumes, and the total scan time was 7 minutes and 45 seconds. A manual specification for the MFC region of interest (ROI) was available for the first data set. For the second data set, 248 healthy subjects (112 male and 136 female; mean age ± SD 25.47 ± 2.27 years) were selected from the HCP data. T1-weighted images were acquired with the following imaging parameters: TR = 2400 ms; TE = 2.14 ms; flip angle = 8°; with voxel dimension = 0.7×0.7×0.7 mm^3^. Imaging parameters for the EPI were as follows: TR = 720 ms; TE = 33.1 ms; flip angle = 52°; 72 slices; with voxel dimension = 2×2×2 mm^3^. EPI data consisted of 1200 volumes, and the total scan time was 15 minutes.

### ROI definition

**Fig 1:**
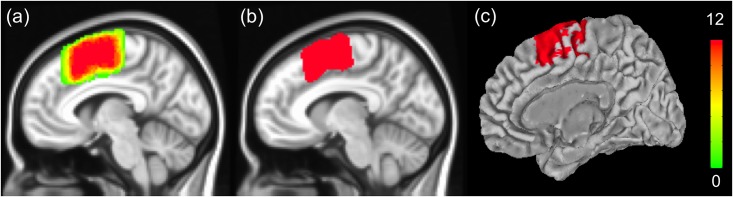
Manual definition of an MFC ROI. (a) Probabilistic MFC ROI. The scale bar denotes the number of cases mapped onto a voxel which ranged from 0 to 12. (b) Probabilistic MFC ROI threshold at 75%. (c) ROI mapped onto the cortical surface.

One of our primary goals was to parcellate the MFC region into sub-regions; therefore, we manually defined the MFC ROI so that subsequent analysis occurred only in the MFC region. For the first data set, an expert neurologist drew the MFC ROIs within the native volume space of a T1-weighted image, which was collected along with the EPI data. Each ROI was later spatially normalized to the Montreal Neurological Institute (MNI) standard volume space using a transformation matrix obtained from routine pre-processing of the fMRI data. Once all 12 ROIs were mapped and summed in the standard space, a threshold of 75% was applied to the summed data to yield a probabilistic MFC ROI. The threshold was chosen as it yielded reasonable results [17]. We computed the probabilistic ROI to reflect group statistics. The probabilistic ROI was then mapped onto the MNI standard surface space. Surface space mapping was necessary because cortical thickness is only available for the surface space, not for the volume space. The defined ROI could be easily applied as it was defined in standard volume and surface space. The procedures and results for defining the ROI in standard surface space are shown in Fig 1.

### FMRI pre-processing

**Fig 2:**
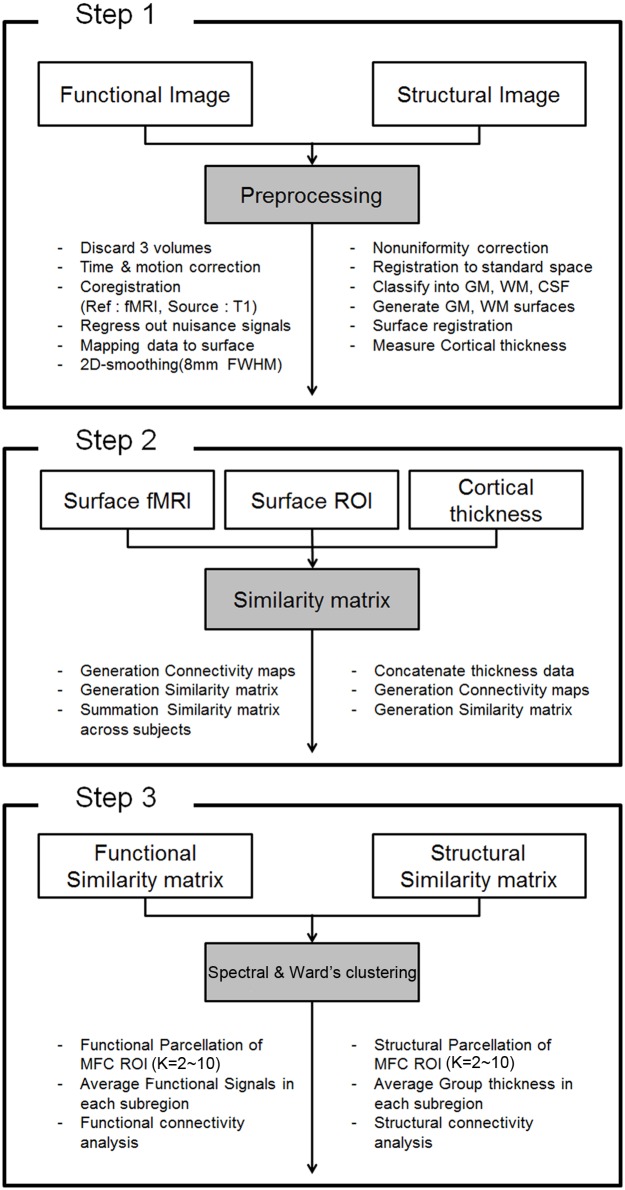
Overview of procedures to parcellate the MFC using cortical thickness and fMRI. TOP: Procedures for pre-processing fMRI and T1-weighted MRI. MIDDLE: Procedures for constructing the similarity matrix. BOTTOM: Procedures for parcellating the MFC regions into k sub-regions using spectral and Ward’s clustering approaches.

Functional MRI data were processed using Analysis of Functional NeuroImages (AFNI) and SUrfaceMApping (SUMA) software [18]. The first three fMRI volumes were removed because they were scanned before magnetization stabilized. The remaining 152 volumes were de-spiked, slice timing-corrected, and realigned for head motion. No participant had head motion greater than 2.0 mm translation or 2 degrees rotation during the resting state scan. A skull-stripped structural T1-weighted image was co-registered to the corrected functional volume. Linear regression was applied to remove nuisance signals unrelated to neuronal activity, including six motion parameters, white matter signals, and ventricular signals. Functional data were temporally band pass filtered (0.009 Hz < *f* < 0.08 Hz) and mapped onto the cortical surface using the two-step two-surface method [19]. First, a straight line consisting of 10 sampling points was drawn between corresponding linked vertices of the gray matter and white matter surfaces. The white matter surface refers to the inner surface between the gray and white matter, while the gray matter surface refers to the outer surface between the gray matter and cerebral spinal fluid (CSF). The time series data were filtered with an 8 mm full width at half maximum (FWHM) 2D Gaussian kernel smoothing. Second, the value of each sampling point was obtained by nearest neighbor 3D interpolation of the filtered time series data. The average value of 10 sample points along the line was assigned as the mapped times series value for the surface vertex. Details can be found in Fig. 4 of [19]. The above procedure is summarized in step 1 of Fig 2.

### Surface extraction and cortical thickness measurement

T1-weighted structural images were processed using automatic image analysis pipeline software developed by the MNI. First, we performed intensity non-uniformity correction. Intensity-corrected brain images were normalized to standard space using a linear transformation. Each subject’s registered brain images were classified into gray matter, white matter, and CSF. The gray and white matter surfaces were extracted using the Constrained Laplacian-Based Automated Segmentation with Proximities (CLASP) algorithm [20,21]. Each subject’s gray and white matter surfaces consisted of 81,924 vertices with triangular meshes. To measure cortical thickness in the respective native spaces, gray and white matter surfaces were inversely transformed to the native spaces because these surfaces were originally extracted from the spatially normalized T1 images. The vertices of gray matter surface maintained correspondence with the vertices of white matter surface. Thus, cortical thickness could easily be measured from the Euclidean distance between corresponding vertices of the gray and white matter surfaces. Cortical thickness maps were then spatially normalized to the standard MNI surface space using a surface registration approach so that cortical thickness could be compared in the common space. In sum, cortical thickness was measured for each subject and then registered onto the standard MNI space using surface registration. Only one registration step was applied to the cortical thickness measurements. Errors in image registration might have caused errors in the mapped thickness map; however, existing studies have adopted the same approach and the associated errors were deemed acceptable, as the registration process caused sub-voxel error [22–24]. The details of this entire procedure are described in a previous paper [22]. The above procedure is also summarized in step 1 of Fig 2.

### Correlation matrix construction

Brain network information is commonly presented in a matrix fashion, where one element of the i-th row and j-th column represents the correlation between the i-th and j-th regions. In this representation, the matrix is referred to as the correlation matrix. Each element of the correlation matrix is a correlation coefficient computed from modality-dependent imaging observations. The first region is commonly referred to as the seed region, as the correlation values with respect to other regions are computed after fixing this first region. The seed region is referred to as the seed vertex in the analysis of the surface space, which we performed in this study. The vertex in this study refers to a vertex on a surface, not a vertex on a graph structure. We limited our study to seed vertices within the MFC since we were interested in how MFC vertices were connected to other brain regions. For the fMRI portion of our study, correlation coefficients were computed for the time series data of a given seed vertex within the MFC and time series data of other cerebral vertices, excluding the MFC. This resulted in a one-row correlation matrix. This process was repeated for all MFC vertices, resulting in an Ns × Nw correlation matrix, where Ns was the number of seed vertices and Nw was the number of other cerebral vertices. The correlation matrix derived from fMRI, denoted as C_fMRI,_ had the following notation. $\;C_{fMRI}\left(i,j\right)=\;\frac{COV\left(X_{i},X_{j}\right)}{\sigma_{X_{i}}\sigma_{X_{j}}}$, where X_i_ and X_j_ were processed fMRI data for vertexes i and j, respectively, COV was the covariance operation, and σ_Xi_ and σ_Xj_ were the standard deviations for X_i_ and X_j,_ respectively [25]. Fisher’s r-to-z transform was applied to correlation values [26]. Functional correlation matrices were summed to yield one correlation matrix to represent a group of subjects. For the thickness portion of this study, the cortical thickness of each subject was concatenated into a long vector and the correlations between concatenated thickness values were used as elements in the correlation matrix. The length of the concatenated vector was the number of subjects being modeled. The pre-processing steps to obtain cortical thickness involved mapping of the cortical thickness values onto the standard surface space; thus, it was possible to concatenate the thickness values at a given vertex location. We computed correlation coefficients between the cortical thickness values of a given seed vertex within the MFC and the cortical thickness values of other cerebral vertices excluding the MFC. This process was repeated for all MFC vertices. The correlation matrix derived from thickness is denoted as C_thick_ with the following notation: $C_{thick}\left(i,j\right)=\;\frac{COV\left(T_{i},T_{j}\right)}{\sigma_{T_{i}}\sigma_{T_{j}}}$, where T_i_ and T_j_ were thickness data for vertexes i and j, respectively, COV was the covariance operation, and σ_Ti_ and σ_Tj_ were the standard deviations for T_i_ and T_j_ respectively. Fisher’s *r*-to-*z* transform was applied to correlation values. One correlation matrix was computed from the cortical thickness values of a group of subjects.

### Inter-subject reliability of correlation matrix

Our study used the sum of correlation matrices across subjects in the fMRI experiment. A 5-fold validation was performed to compute the stability of connectivity patterns. HCP data were randomly assigned to five groups of 50, 50, 50, 50, and 48 subjects. The sum of 198 correlation matrices from the four groups was compared against the sum of 50 correlation matrices from the left-out group for each MFC vertex. Comparisons were made using Pearson’s correlation coefficient. This process was repeated 30 times, each time with a different set of 50 subjects as the left-out group. Vertex-wise correlation maps were then averaged across leave-one-out steps to yield the average stability map. We applied the same 5-fold validation approach to HCP data to compute the inter-subject reliability for thickness-driven connectivity. HCP data were randomly assigned to five groups. Each group had 50 or 48 thickness measurements that were used to compute one correlation matrix, as described in “Correlation matrix construction”. The sum of four correlation matrices from the four groups was compared against the correlation matrix for the left-out group for each MFC vertex. This process was repeated 30 times, each time with a different set of 50 subjects as the left-out group. Vertex-wise correlation maps were then averaged across leave-one-out steps to yield the average stability map.

### Parcellation using clustering

We applied spectral clustering and Ward’s clustering algorithms with a variable number of clusters (i.e., values between two and 10) to all MFC vertices [8,27–29]. Spectral clustering considers the eigen-spectrum of the correlation matrix and is more capable of producing non-convex decision boundaries than the traditional k-mean clustering approach [15]. Spectral clustering was chosen as it is a widely used clustering approach [27]. The affinity matrix (A) for spectral clustering was generated by computing pair-wise correlations between rows of the correlation matrix. A transform of adding a constant of one and then dividing by two was applied to correlation values to ensure non-negativity in the affinity matrix. For example, an element in the i-th row and j-th column from A would be the correlation between the i-th row of the correlation matrix and the j-th row of the correlation matrix. Ward’s clustering is a hierarchical algorithm where each data point belongs to a hierarchy of clusters. Data points with similar properties were merged using a criterion such as minimum-variance. Ward’s clustering was adopted as it was shown to perform best among three tested clustering algorithms in a recent parcellation study [30]. Dimensionality reduction was not applied for the Ward’s approach. Clustering algorithms were applied twice, once to the fMRI correlation matrix and then again to the cortical thickness correlation matrix obtained from the HCP data (n = 248). Each vertex within the MFC was assigned to pre-determined k clusters. This procedure essentially parcellated the MFC into k sub-regions based on the given correlation matrix. The above procedure is also summarized in step 3 of Fig 2.

### Determining the number of clusters: fMRI

The MFC was parcellated into k sub-regions, where k was a user-defined parameter. Variation of information (VI) was adopted to determine the k value to use [31]. A split-half comparison was performed by randomly assigning subjects into two groups of 124 subjects. The summed correlation matrix for each group was used to yield corresponding clustering results. The two sets of clustering results were compared using the VI metric for a given k. This procedure was repeated 100 times, each time with new random split-half groups. Low VI values indicated high similarity between the two clustering results (i.e., high stability), while high VI values indicated low similarity (i.e., low stability). A plot of average VI values across split halves was prepared as a function of k. The trend of VI value with respect to k was used to determine the optimal number of clusters to use. One possible criterion was to determine k such that VI did not decrease substantially relative to the VI of k-1 clusters [31]. This approach can be thought of as choosing a k that leads to a plateau in VI values, starting from the largest k. A silhouette reproducibility approach was adopted to determine which k value to use [32]. Silhouette coefficients measured how close each point in one cluster was to points in neighboring clusters, which could be used to determine how many clusters to use. For each subject, parcellation was performed using k clusters and results were used to compute a silhouette coefficient. We computed the mean silhouette value. The optimal number of clusters could be chosen where maximum silhouette value occurred.

### Whole brain network analysis with respect to MFC

In the previous sub-section, we analyzed the brain network for all vertices within the MFC region. In this sub-section, we analyzed the whole brain network using the k sub-divided MFC regions as the seed region while exploring other brain regions from the second data set (n = 248). The sub-divided MFC region as a whole (i.e., not individual vertices) in each hemisphere was correlated with other brain regions. We adopted the Automated Anatomical Labeling (AAL) atlas defined on the common MNI space to designate various brain regions [33]. For the fMRI portion of our study, we computed the spatial average of time series data in the sub-divided MFC region and other regions obtained from the AAL atlas. For each participant, correlation coefficients between time series data were computed by fixing the seed region and exploring the other regions, which yielded a correlation matrix. We applied one-sample t-tests to correlation values of regions and retained regions whose false discovery rate (FDR) corrected p-values were less than 0.05. For the thickness portion of this study, we computed the spatial average of group-wise cortical thickness values in the sub-divided MFC regions and other regions from the AAL atlas obtained from the 248 participants. Correlation coefficients between cortical thickness values were computed by fixing the seed region and exploring the other regions, which yielded a correlation matrix. We identified significant regions as those with z-values (i.e., normalized correlation values) higher than 0.4. The *z* value was assumed to follow the normal distribution with a mean of zero and a standard error of $1/\sqrt{n-3}$, where n was the number of subjects [26].

## Results

### Stability of the connectivity pattern

**Fig 3:**
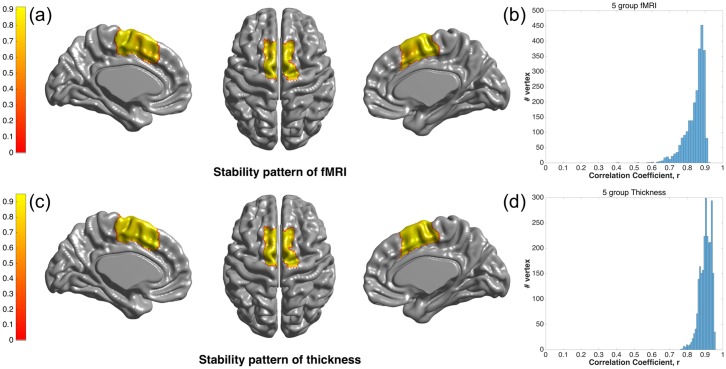
Stability of connectivity patterns. (a) Map of correlation coefficients describing the average correlation between MFC cortical thickness-driven connectivity patterns (between activity in the MFC and the rest of the brain). (b) Histogram of correlation coefficients between the MFC connectivity patterns of different subjects based on thickness. (c) Map of correlation coefficients describing the average correlation between MFC fMRI-driven connectivity patterns (between activity in the MFC and the rest of the brain). (d) Histogram of correlation coefficients between the MFC connectivity patterns of different subjects based on fMRI.

The stability of fMRI- and thickness-driven connectivity patterns within the MFC was investigated to provide a basis for using a summed correlation matrix to represent a group of subjects. If the stability of a connectivity pattern was low, it suggested that the subjects had diverging connectivity patterns. This would be a weak basis for using group statistics for parcellation. The average stability in terms of the correlation coefficient was 0.85 for fMRI and 0.9 for cortical thickness. The average stability map was plotted along with the distribution of correlation coefficients in Fig 3. Correlations greater than 0.7 were observed throughout the MFC vertex except for boundary vertices.

### Determining the number of clusters: fMRI

**Fig 4:**
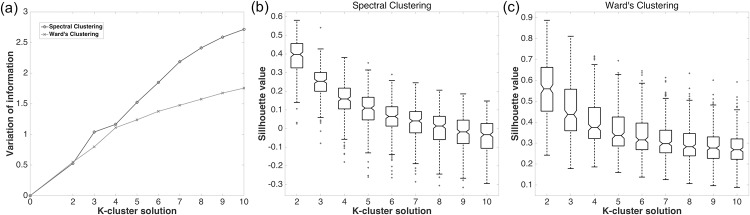
Choosing the number of clusters. (a) Plot of VI values with respect to the number of clusters (k = 2..10) for two clustering algorithms (spectral and Ward’s clustering approaches). All VI values at different k values were statistically significant (p < 0.05). There was no plateau as we decreased k from 10 to 2. (b) Plot of silhouette coefficient with respect to the number of clusters (k = 2..10) for the spectral clustering approach. (c) Plot of silhouette coefficient with respect to the number of clusters (k = 2…10) for Ward’s clustering approach.

This study parcellated the MFC into k sub-regions, where k was a user-defined parameter. The VI metric was adopted to determine what k values to use. A plot of average VI values across split-halves was presented as a function of k (Fig 4). The VI values increased significantly (p < 0.05) with respect to all k values between 2 and 10. Starting from large k values (= 10), we could not find the smallest k that resulted in a non-significant decrease in VI value for both clustering algorithms. The VI metric did not lead us to an optimal k, thus we chose the smallest number (i.e., k = 2) as the number of clusters. Given a choice of k values, the model with the fewest degrees of freedom was typically the most suitable [17]. A plot of average silhouette values was given as a function of k (Fig 4). Using k = 2 led to the highest silhouette value. The use of two clusters was consistent with the existing literature, as the MFC is commonly believed to have two distinct sub-regions [1]. We also used two clusters in the cortical thickness portion of our study in order to produce a fair comparison between fMRI and cortical thickness results.

### Functional and structural MFC parcellation

**Fig 5:**
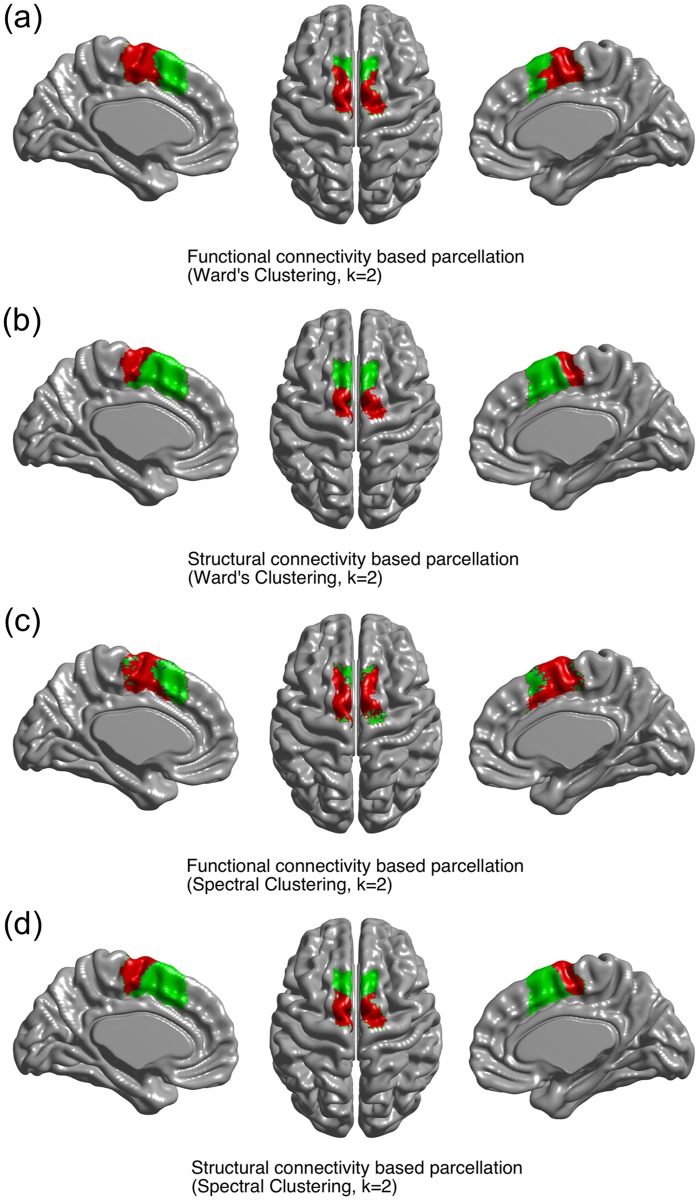
Parcellation results using (a) connectivity from fMRI and (b) connectivity from cortical thickness based on Ward’s clustering with K = 2. Parcellation results using (c) connectivity from fMRI and (d) connectivity from cortical thickness based on spectral clustering with K = 2. The anterior portion is shown in green for the pre-SMA and the posterior portion is shown in red for the SMA.

We performed parcellation of all vertices in the MFC regions into two (k = 2) clusters, first using the fMRI-driven correlation matrix and then using the cortical thickness-driven correlation matrix. Parcellation based on fMRI data revealed two distinct sub-regions in both the left and right hemispheres, corresponding to the anterior part (i.e., pre-SMA) and the posterior part (i.e., SMA), as shown in Fig 5. Parcellation based on cortical thickness data also revealed two similar-looking distinct sub-regions within the MFC, as shown in Fig 5. Visual inspection revealed agreement between fMRI-based and thickness-based parcellation results. Consistent with visual inspection, dice overlap index values between the two parcellation results were 0.47 for the pre-SMA and 0.60 for the SMA using spectral clustering. The dice overlap index values were 0.74 for the pre-SMA and 0.75 for the SMA using Ward’s clustering. Agreement of parcellation using fMRI- and thickness-driven connectivity yielded average dice overlaps of 0.74 (Ward’s clustering) and 0.54 (spectral clustering) for two sub-regions in the MFC. These performance values reinforced the consistency between the two sets of parcellation results.

### Comparison with Brodmann atlas

**Fig 6:**
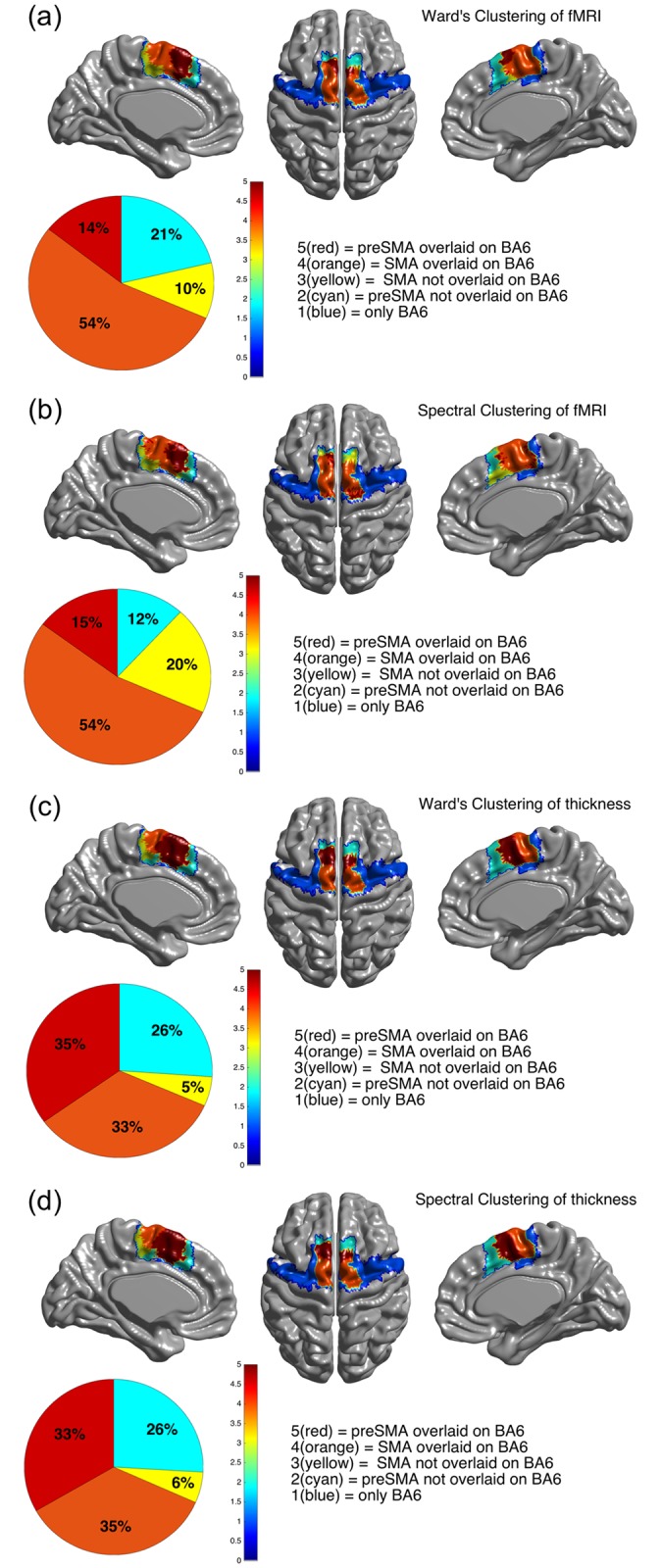
Overlap between BA6 and parcellation results using two clustering approaches based on two types of connectivity. (a) Using fMRI and Ward’s clustering. (b) Using fMRI and spectral clustering. (c) Using thickness and Ward’s clustering. (d) Using thickness and spectral clustering.

Parcellation results using two clustering algorithms were compared with the Brodmann atlas to assess agreement between our parcellation results and Brodmann atlas cytoarchitecture. Our parcellation results were compared with Brodmann area (BA) 6. The fMRI- and thickness-driven parcellation results were overlaid with BA 6 of the HCP Brodmann atlas in Fig 6. Our parcellation results were mainly contained within BA 6. Approximately 70% of MFC sub-regions (i.e., SMA and preSMA) overlapped with BA6, as shown in red and orange in Fig 6. The amount of overlap did not change much, regardless of whether we adopted fMRI- or thickness-driven connectivity. The amount of overlap did not change based on Ward’s or spectral clustering.

### Whole brain network analysis

**Fig 7:**
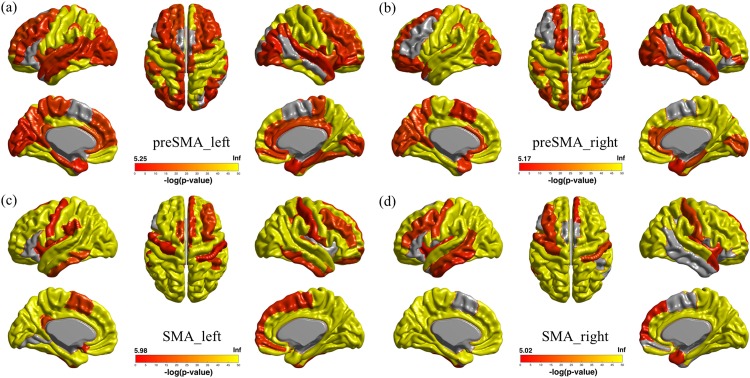
fMRI-driven connectivity results of the whole brain using the MFC sub-region as the seed region. (a) Connectivity results using the left pre-SMA (i.e., anterior) region as the seed region. (b) Connectivity results using the right pre-SMA region as the seed region. (c) Connectivity results using the left SMA (i.e., posterior) region as the seed region. (d) Connectivity results using the right SMA region as the seed region. Colored values denote negative log of p-values.

We analyzed the whole brain network of the fMRI time series data using the sub-divided MFC regions as seed regions and exploring other brain regions, as shown in Fig 7. The pre-SMA and SMA regions were parcellated using Ward’s clustering results as they yielded a better overlap index than spectral clustering. One analysis was performed using the pre-SMA (i.e., anterior) region as the seed region, and the other analysis was performed using the SMA (i.e., posterior) region as the seed region. The SMA region was significantly (corrected *p* < 0.00001) correlated with motor regions consisting of the precentral gyrus and medial cingulate cortex. In contrast, the pre-SMA region was less correlated with these motor regions. The pre-SMA region was correlated (corrected *p* < 0.00001) with frontal regions that consisted of the superior frontal gyrus and the medial frontal gyrus. The SMA region was less correlated with these same frontal regions. Functional connectivity results with respect to the SMA and pre-SMA regions are shown in Table 1.

**Table 1:** Major correlated regions in the motor and frontal areas for SMA/pre-SMA regions using fMRI data. Regions with significance are reported in bold.

Sub-region	Target region	Left/Right	One sample *t*-test (*t*-value)	Corrected *p*-value
Left SMA	**Precentral**	**Left**	10.8319290082795	**< 0.00001**
		**Right**	9.88344268953952	**< 0.00001**
	**Mid-Cingulate**	**Left**	12.4780727572292	**< 0.00001**
		**Right**	13.5169888816289	**< 0.00001**
	**Angular**	**Left**	12.7936976481766	**< 0.00001**
		**Right**	9.89283126920642	**< 0.00001**
Right SMA	**Precentral**	**Left**	18.8483788304059	**< 0.00001**
		Right	-1.45981924853788	**0.92719**
	**Mid-Cingulate**	**Left**	25.909367603036	**< 0.00001**
		**Right**	17.5317277429267	**< 0.00001**
	**Angular**	**Left**	16.5996055376089	**< 0.00001**
		**Right**	21.5147665722438	**< 0.00001**
Left pre-SMA	**Superior Frontal**	**Left**	15.000356670372	**< 0.00001**
		**Right**	10.0795534334249	**< 0.00001**
	**Medial Frontal**	**Left**	6.50825043771273	**< 0.00001**
		**Right**	3.01904147004437	**0.00140**
	**Angular**	**Left**	7.16356089807129	**< 0.00001**
		**Right**	9.98370029717117	**< 0.00001**
Right pre-SMA	**Superior Frontal**	**Left**	12.7449899471104	**< 0.00001**
		**Right**	9.52338949936237	**< 0.00001**
	**Medial Frontal**	**Left**	7.37536786913104	**< 0.00001**
		**Right**	6.4325149682644	**< 0.00001**
	**Angular**	**Left**	10.88629793357	**< 0.00001**
		**Right**	9.46581707369649	**< 0.00001**

**Fig 8:**
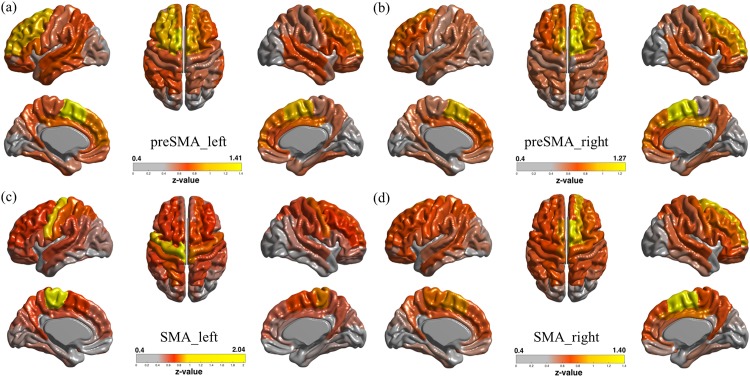
Cortical thickness-driven network analysis of the whole brain using MFC sub-regions as the seed region. (a) Connectivity results using the left pre-SMA (i.e., anterior) region as the seed region. (b) Connectivity results using the right pre-SMA region as the seed region. (c) Connectivity results using the left SMA (i.e., posterior) region as the seed region. (d) Connectivity results using the right SMA region as the seed region. Colored values denote z-values.

We also analyzed the whole brain network of cortical thickness data using the sub-divided MFC regions as the seed regions and exploring other brain regions, as shown in Fig 8. One analysis was performed using the pre-SMA (i.e., anterior) region as the seed region, and the other analysis was performed using the SMA (i.e., posterior) region as the seed region. The SMA region was significantly (z > 0.4) correlated with motor regions consisting of the precentral gyrus and medial cingulate cortex, as reported in Table 2. This result from cortical thickness data was also consistent with that from the fMRI connectivity data. In addition, the pre-SMA region was significantly (*z* > 0.4) correlated with frontal regions that consisted of the superior frontal gyrus and medial frontal gyrus, as reported in Table 2. This result from cortical thickness data was also was consistent with that from the fMRI connectivity data. Overall, whole brain network analyses using the MFC sub-regions as the seed regions were consistent, whether we used fMRI or cortical thickness data. We also observed that, even though regions showing major correlation were consistent for fMRI connectivity- and cortical thickness-based correlation maps, there were differences, including the connection strength between regions. In general, the whole brain network information derived from fMRI and cortical thickness was similar for the MFC region.

**Table 2:** Major correlated regions (motor and frontal regions) for SMA/pre-SMA regions using cortical thickness. Regions with significance are reported in bold.

Sub-region	Target region	Left/Right	Correlation Coefficient (*z*-score)	Corrected *p*-value
Left SMA	**Precentral**	**Left**	0.914888246	**< 0.00001**
		**Right**	0.778042883	**< 0.00001**
	**Mid-Cingulate**	**Left**	0.609910529	**< 0.00001**
		**Right**	0.575345189	**< 0.00001**
	**Angular**	**Left**	0.570550540170045	**< 0.00001**
		**Right**	0.481691049404179	**< 0.00001**
Right SMA	**Precentral**	**Left**	0.702214913	**< 0.00001**
		**Right**	0.751713666	**< 0.00001**
	**Mid-Cingulate**	**Left**	0.646114132	**< 0.00001**
		**Right**	0.815058405	**< 0.00001**
	**Angular**	**Left**	0.644203779344561	**< 0.00001**
		**Right**	0.559207603830776	**< 0.00001**
Left pre-SMA	**Superior Frontal**	**Left**	0.999544391	**< 0.00001**
		**Right**	0.88498706	**< 0.00001**
	**Medial Frontal**	**Left**	0.931279677	**< 0.00001**
		**Right**	0.7777322	**< 0.00001**
	**Angular**	**Left**	0.673914948196877	**< 0.00001**
		**Right**	0.518119231076202	**< 0.00001**
Right pre-SMA	**Superior Frontal**	**Left**	0.788382444	**< 0.00001**
		**Right**	1.06106518	**< 0.00001**
	**Medial Frontal**	**Left**	0.72027127	**< 0.00001**
		**Right**	0.843095283	**< 0.00001**
	**Angular**	**Left**	0.581874154230906	**< 0.00001**
		**Right**	0.475966583265475	**< 0.00001**

## Discussion

This study is the first to compare network information derived from fMRI and cortical thickness. We observed that both fMRI- and cortical thickness-based parcellation results divided the MFC into two sub-regions that corresponded to the pre-SMA and the SMA regions. The fMRI parcellation results and those for cortical thickness were consistent and showed high overlap between the two parcellation results. This result led us to believe that 1) two distinct sub-regions, the SMA and pre-SMA regions, have sharp differences in terms of functional connectivity and morphological inter-regional correlation and thus justified parcellation into two distinct sub-regions, and 2) morphological inter-regional correlation was strongly linked with functional connectivity within the MFC region. In the MFC sub-regions (i.e., the pre-SMA and the SMA), functional divisions based on fMRI and structural divisions based on cortical thickness might be very similar; however, this might not apply for many other brain regions.

This study also compared whole brain network analysis using MFC sub-regions as seed regions and exploring other brain regions using both fMRI and cortical thickness data. The SMA region showed strong functional correlations with the precentral gyrus and medial cingulate cortex. This is consistent with existing findings showing that the SMA is closely associated with motor control [34–37]. Unlike the SMA, the pre-SMA region showed strong functional correlations with most of the frontal region. The observed functional connectivity of the pre-SMA closely resembled that for complex cognitive controls, which include alteration of motor plans, motor selection, and sequential movement [37–39]. Additionally, based on cortical thickness-driven correlation maps, the SMA region showed strong correlations with motor regions, and the pre-SMA region showed strong structural correlations with most of the frontal region. We conducted whole brain network analyses using the same approach adopted to parcellate the MFC into sub-regions and confirmed the known findings. Thus, we believe the approach we adopted for MFC parcellation is sound.

Our study belongs in the category of network analyses of brain connectivity. Recent studies have adopted cortical thickness to explore brain networks and have demonstrated an intrinsic property of the whole brain network, namely “small-worldness” of the structural network and a modular architecture property of the whole brain [13,40]. Both results demonstrated that the anatomical network in the human brain was largely compatible with the previously identified functional network. Using independent component analysis, another study demonstrated that gray matter structural components were closely associated with functional components [41]. In light of these previous findings, our results suggest that inter-regional correlation maps derived from cortical thickness reflect the underlying cytoarchitecture and function of some brain regions.

We applied two types of clustering algorithms, spectral and Ward’s clustering. As shown in a recent study, Ward’s clustering was better than k means and spectral clustering [30]. A good clustering algorithm should be able to produce consistent parcellation results if two types of connectivity share a similar connectivity profile for the MFC. Indeed, Ward’s clustering provided better agreement between fMRI- and thickness-driven parcellation results than spectral clustering. We adopted the AAL atlas, a structural atlas, for whole brain connectivity analysis. Using a functional atlas is better suited for a whole brain functional connectivity study and should be considered for future studies. One major advantage of using the AAL is that it is a widely adopted atlas, so results derived from the AAL atlas can be easily compared across studies. Two types of network information led to consistent whole brain connectivity results in the medial frontal gyrus and superior frontal gyrus, regions related to working memory [42,43]. We observed inconsistent whole brain connectivity results in the angular gyrus (responsible for language and spatial cognition) based on two types of network information [44]. Further investigation is needed to understand why two types of network information agree for working memory-related regions. Our approach was entirely performed on the cortical surface and thus is only applicable to brain regions that are close to the cortical surface. Certain deep brain structures far from the cortical surface cannot be analyzed with the approach here and will require further investigation.
